# Perceptions and practices on substandard and falsified medicines in humans and animals in Wakiso district, Uganda: A qualitative study

**DOI:** 10.1371/journal.pone.0339569

**Published:** 2025-12-26

**Authors:** David Musoke, Grace Biyinzika Lubega, Claire Brandish, Kate Russell-Hobbs, Natasha Hamilton, Jody Winter, Carol Esther Nabbanja, Filimin Niyongabo, Michael Obeng Brown, Elma Rejoice Banyen, Herbert Bush Aguma, Linda Gibson

**Affiliations:** 1 Department of Disease Control and Environmental Health, Makerere University School of Public Health, Kampala, Uganda; 2 Pharmacy Department, Buckinghamshire Healthcare NHS Trust, Aylesbury, United Kingdom; 3 Department of Biosciences, School of Science and Technology, Nottingham Trent University, Nottingham, United Kingdom; 4 Institute of Health and Allied Professions, School of Social Sciences, Nottingham Trent University, Nottingham, United Kingdom; 5 Department of Pharmacy, School of Health Sciences, Makerere University College of Health Sciences, Kampala, Uganda; Shanghai Jiao Tong University, CHINA

## Abstract

**Background:**

Few studies have taken a broad perspective on substandard and falsified medicines (SFMs) in community settings in Uganda. We therefore qualitatively assessed the perceptions and practices on SFMs for humans and animals in Wakiso District, Uganda.

**Methods:**

This qualitative study employed 12 focus group discussions among community health workers and farmers, as well as 11 key informant interviews among health professionals, local leaders, veterinary and human drug shop operators, and Ministry of Health and Ministry of Agriculture, Animal Industry and Fisheries officials. Data was analysed thematically using NVivo (2020).

**Results:**

Findings are presented under four themes: definition of SFMs; identification of SFMs; drivers of SFMs; and challenges in reporting SFMs. Although participants felt that the term SFMs was too broad to define, many explained it in relation to consequences (such as side effects, disabilities, and death), and the different methods that may be employed to identify SFMs. SFMs were said to be identified through changes in appearance such as colour, texture and packaging. However, the most reported way of identifying SFMs was based on negative effects on humans and animals after use. Customer drivers of SFMs included inadequate knowledge, lack of finances, and lack of access to licensed drug shops/ pharmacies. Supplier drivers of SFMs included limited regulation and enforcement, and the business orientation of drug shops/ pharmacies. Lack of knowledge of how to report suspected cases of SFMs, lack of proof of purchase of SFMs, fear of reporting, and lack of trust in government procedures were the challenges identified to reporting SFMs.

**Conclusion:**

Several drivers of SFMs in the community were established related to individuals, sources of medicine, and regulatory frameworks. Increased awareness on SFMs, improved traceability of purchases and reporting, and better enforcement of regulations are needed to reduce the use and risks associated with SFMs to protect public health.

## Introduction

Substandard medicines are authorised medical products that fail to meet the quality standards or specifications set out by regulatory bodies [1,2]. They may contain insufficient quantities of active ingredients or incorrect formulations, typically due to deficiencies in the manufacturing, distribution and/or storage processes [1,2]. For example, the quality of a medicine can deteriorate over time if it is stored incorrectly, leading to inadequate dosing. Falsified medicines deliberately misrepresent their identity, composition, and/or source. These products may intentionally contain no active ingredients, wrong ingredients, or incorrect quantities of the ingredients [1,2]. Between 2017 and 2021, the global incidence of Substandard and Falsified medicines (SFMs) increased by 36.3% [3]. SFMs have been identified as potential drivers of antimicrobial resistance (AMR) [4–9], undermining antimicrobial stewardship (AMS) efforts. As such, action plans, including the Uganda AMR National Action Plan, have a strong emphasis on strengthening detection mechanisms to identify and address SFMs [10,11].

Despite SFMs being a global problem, counterfeit pharmaceuticals have an estimated global trade value of 4.4 billion USD per year [12], driven in part by online availability [13,14], and limited access to quality health care services, especially in low- and middle-income countries (LMICs) [3]. The World Health Organization (WHO) estimates that one in ten medical products in LMICs is substandard or falsified [1] hence, the African continent bears the largest burden (>75%) of SFMs alerts [15]. Recent systematic reviews estimated that 18.7–22.6% of medicines in Africa [16,17], 26.9% in West and Central Africa [18], and 22.6% in East Africa [7] are SFMs. SFMs were responsible for approximately 122,350 under‑5 deaths across 39 sub‑Saharan countries [19], 693 under‑5 in Benin [20] and 12,300 in Nigeria [21] annually.

Through the support of the African Union, the African Medicines Regulatory Harmonization Initiative (AMRH) was established to increase access to safe and effective medicines in 2009 [22,23]. The AMRH initiative led to the establishment of the African Medicines Agency (AMA) in 2019, which aims to ensure that all Africans have access to affordable medical products for priority diseases/conditions that meet internationally recognized standards of quality, safety and efficacy [22–24]. In East Africa, the regulation of medical products is through the respective national medicines regulatory authorities supported by East African Community regulatory harmonisation initiative, which has contributed to better control of SFMs [25]. Furthermore, Uganda is part of the Lomé Initiative, which was signed amongst 7 countries to criminalise the trafficking of SFMs in Africa [15,26]. Within Uganda, National Drug Authority (NDA) is the country’s National Medicines Regulatory Authority (NMRA) and is therefore responsible for the regulation (quality, safety and efficacy) of all pharmaceutical products in human and animal health [27,28]. Despite the availability of these agencies, identification, monitoring and reporting of SFM remains a major challenge in most LMICs, including Uganda [24,29,30].

The structure of Uganda’s pharmaceutical sector and health supply chain has been described elsewhere [31–34]. This sector in the country is allocated 14% of the health budget, which is only 7.8% of the national budget [35]. Additionally, 96% of its pharmaceutical products are imported [36]. The government has made strides to expand local pharmaceutical production (LPP) by imposing import tariffs on imported pharmaceuticals, which has increased 8.2% of LPP [37]. Despite a 15.9% increase in essential medicines and health supplies, the overall allocation of resources remains low to meet both public and private health needs [38]. In Uganda, the most commonly available SFMs are for lifestyle conditions (such as diabetes and hypertension), impotence, malaria and bacterial infections [39–42]. Poor quality antimalarial drugs have been estimated to cost Ugandan patients $26 million each year, with the health and economic burden mainly on poor and rural populations, furthering health inequities [41]. Application of the Substandard and Falsified Antimalarial Research Impact (SAFARI) model in Uganda found that 9% of malaria deaths in people who had sought treatment were attributable to poor quality antimalarial drugs [43]. A recent study in Ghana, Nigeria, Sierra Leone and Uganda found that less privileged groups were disproportionately exposed to SFMs and had lower awareness of them [44]. Over 30% of respondents in Uganda reported having obtained medicines from unofficial sources, most commonly, street hawkers and family/friend [44]. A recent assessment of the pharmacopeial quality of artemether-lumefantrine antimalarials in Uganda found that all samples passed visual inspection but 18.9% of the samples were of substandard quality in terms of their drug content [45].

The drivers, prevalence and impacts of substandard medicines have been relatively well-studied. These drivers include limited access to affordable, safe and high quality medicines, limited in-country manufacturing capacity with quality assurance systems, weak regulatory systems, poor governance and enforcement including weak reporting mechanisms, poor drug distribution practices, poor drug supply chain management leading to drug stock outs, presence of crime entities, and limited public awareness [3,15,46]. The impacts of SFMs can be summarised into three categories: health (increased morbidity and mortality, progression of AMR, higher disease prevalence, loss of consumer trust in modern medicines, health workers and health systems, increased risk of side effects, and adverse reactions); socioeconomic (loss of income and productivity, increased poverty, and lack of social mobility); and economic impact (economic loss, increase out of pocket spending, and wastage of resources) [15,31,47]. Qualitative studies have been employed to understand the problem of SFMs with views from multiple stakeholders in sub-Saharan Africa [48–54]. However, most of these studies have a focus on human health [55], which creates a knowledge gap for understanding SFMs and the implications of this for AMR using One Health approaches [56]. To fill in this gap, we undertook an in-depth exploration of experiences and perceptions of stakeholders from a One Health perspective on SFMs in the Ugandan context. These stakeholders included representatives from the human health, animal health, and environment sectors. This study, therefore, qualitatively assessed the perceptions and practices on SFMs for use in humans and animals in Wakiso District, Uganda.

## Methodology

### Study design, participants and setting

This was a qualitative study that employed Focus Group Discussions (FGDs) among community health workers (CHWs) and farmers, as well as key informant interviews (KIIs) among health professionals, local leaders, veterinary and human drug shop operators, and personnel from the Ministry of Health (MOH) and Ministry of Agriculture, Animal Industry and Fisheries (MAAIF). Participants in this study were selected with respect to the Uganda AMR National Action Plan (NAP) that emphasises the importance of employing a One Health approach when conducting research on AMR, given that this local and global challenge has no sectoral boundaries [57]. Additionally, having participants from both human and animal health sectors ensured that diverse perspectives were obtained which could be integrated into unified policy recommendations. This study was conducted in peri-urban and rural areas of Kakiri Town Council and Sub County, Masuulita Town Council and Sub County, and Namayumba Town Council and Sub County in Wakiso district. Wakiso district was chosen for the study because it is the most populated district in Uganda with extensive rural, peri urban and urban areas. Wakiso District is situated in Uganda’s central region and partially surrounds the capital city of Uganda. The district has a population of 3,397,555, [58] and is divided into 2 counties (Kyadondo and Busiro). These counties are further divided into 8 constituencies, 8 sub counties, 8 town councils, 148 parishes, and 704 villages.

### Sampling

A total of 12 FGDs and 11 KIIs were conducted. This sample size was arrived at when data saturation was reached during data collection. The 12 FGDs were conducted in the selected rural, peri-urban and urban villages of Namayumba Subcounty and Kakiri Town. These areas were purposively selected because of being remote yet they had a reasonable number of veterinary drug shops and private pharmacies. CHWs who were actively involved in health promotion, and farmers who were practicing animal husbandry were purposively selected to participate in the FGDs after consultations with village leaders and mobilisers. Each FGD comprised of 8–12 participants, and a variety of male only, female only, mixed sex, and youth groups were constituted. This strategy was employed because mixing respondents could have affected the quality of the discussions considering some cultural, gender-related beliefs in the society. Some FGDs combined CHWs and farmers to explore how health and agricultural practices and knowledge on SFMs can influence each other in rural communities. The FGDs were carried out in four different categories (Table 1).

**Table 1:** Focus Group Discussion categories.

Category	CHWs	Farmers	CHWs and farmers	Total
Male only	1	1	1	**3**
Female only	1	1	1	**3**
Youth	1	1	1	**3**
Male and female	1	1	1	**3**
**Total**	**3**	**3**	**3**	**12**

All of the 11 key informants were purposively selected based on their expertise and relevance to SFMs and included representatives from Wakiso District Local Government (2), MOH (2), and MAAIF (2), Veterinary drug shop operators (2), human health drug shop operators (2), and 1 local council leader.

### Data collection

The qualitative data collection tools (FGD and KII guides) explored perceptions and practices in relation to SFMs use in humans and animals in community setting. Data was collected between 1^st^ April to 31^st^ May 2024 using an FGD guide in *Luganda*, while the KIIs were conducted using a guide either in English or *Luganda* (depending on the participants). Both the FGD and KII guides covered several topics in relation to SFMs used for both humans and animals including definition, identification, availability, practices, consequences, reporting, and interventions. The guides were developed with reference to existing literature from previous research [49,50,59]. The guides were pre-tested among households in Mulago cell in Kampala to check for consistency, clarity and eliminate any errors. Research Assistants (RAs) who had bachelors degrees were trained and oriented by the Principal Investigator to ensure they were well versed with the tools before data collection. The RAs were also equipped with various interviewing skills such as probing and the appropriate way of recording responses. It was ensured during the training that the RAs understood the study aims and methodology. A research supervisor ensured that all information was accurately collected and recorded during the data collection process.

For the FGDs, 1 RA moderated the sessions while another assisted with voice recording and note taking. Each FGD lasted approximately 75 minutes and were carried out in a quiet place of the selected villages in the sub counties or town councils as guided by the CHW coordinators. For the KIIs, each RA interviewed a participant for approximately 30 minutes while recording the interview session and writing notes. Each of the KIIs were undertaken after setting an appointment with the participant based on their availability.

### Data management and analysis

Qualitative data from the FGDs and KIIs was audio recorded and transcribed verbatim by the RAs. For data analysis, both the FGD and KII transcripts were read more than 3 times by researchers (GBL, FN and MOB). Thereafter, the researchers met and discussed the key words or related phrases from the transcripts, which were then grouped to form more defined codes. Using NVivo (2020) software, the agreed codes were used to generate a code book. From the code book, the wider team (GBL, MOB, FN and DM) met and discussed the themes and subthemes that were generated from the study.

### Ethical considerations

Ethical approval (MAKSHSREC-2023–607) was obtained from Makerere University College of Health Sciences School of Health Science Research and Ethics Committee. The research was also approved and registered (HS3736ES) at the Uganda National Council for Science and Technology (UNCST). Participation in the study was voluntary, and participants provided written informed consent after they received an explanation of the proposed research including the anticipated risks and potential benefits of taking part. All data emanating from the study was confidentially stored to limit access.

## Results

Findings from the study are presented under 4 themes: definition of substandard and falsified medicines (SFMs); identification of SFMs; drivers of SFMs; and challenges in reporting SFMs.

### Definition of substandard and falsified medicines

Although FGD participants mentioned that the term SFMs was too broad for them to define, many explained SFMs in relation to their consequences (such as side effects, disabilities, death, deterioration, or no change in health condition), while others used the different methods that may be employed to identify SFMs (such as colour, texture and packaging, and expiry dates) to define them.

*“In my opinion, a falsified medicine is one that cannot do what it is supposed to do in the expected way. Or it could be worsening my condition whenever I take it to heal me from an illness.*” **Participant 05, Male only CHW FGD 08**

Among the key informants, substandard medicines were drugs with technical inefficiencies which meant that their active ingredients did not meet the approved measurements. Falsified medicines were defined as those lacking an active ingredient required to provide a desired effect for a particular condition. Additionally, key informants mentioned that some drugs become substandard due to poor handling and storage conditions.

*“Sometimes substandard medicines are due to either deliberate or technical inefficiencies in the manufacturing such that the active ingredients do not meet the desired measurements, or if the chemicals were of the right quantities, you find that the handling practices like the packaging or transportation degrade the quality of that medicine. Then, falsified medicines are reported to having curative properties for certain conditions when in actual sense they do not have the capacity to cure such conditions.”* **Key Informant 02, Veterinary personnel**

### Identification of substandard and falsified medicines

FGD participants identified SFMs in 2 main ways: their appearance and consequences. Regarding appearance, a medicine was considered substandard or falsified if it had no quality mark, seal, batch number, label, or packaging. Some participants mentioned that if medicines had been tampered with, as indicated by a broken seal or a difference in the spelling of words on the packaging, they would consider them substandard or falsified. Other participants also mentioned changes in the colour and texture of the actual medicine as key features for the identification of falsification or being substandard. Checking the expiry date was the most frequently mentioned means of identification of substandard drugs, despite many CHW FGD participants saying that this process was rarely undertaken.

*“Falsified medicine does not have a trademark and specifications that you can follow. When some people make falsified medicines, they put them in disposable bottles. I can understand falsified medicine in different ways: it does not have clear usage instructions, has no expiry date, it has no manufacturer and distributor information.”* **Participant 11, Male only, Mixed CHW and Farmers, FGD 06, Namayumba Town Council**

The most common method of recognising whether medicines were substandard or falsified was through observation of the consequences in humans and animals after their use. FGD participants mentioned several outcomes including vomiting, poisoning, allergic reactions, disabilities, prolonged and deteriorating illness, other side effects, and in worst-case scenarios death. Despite participants mentioning the different consequences of SFMs, many of them testified that they could only distinguish SFMs after experiencing their effects. Without the actual use of the medicine, participants said that it was difficult to distinguish between genuine and fake medicines. This same view was also echoed among the key informants as many highlighted that most community members realised the medicines they had purchased were substandard or falsified through their effects, or lack of them, for example failure to cure their illness.

*“On my own, I would say it is not easy to identify that a medicine is falsified unless you first use it and do not recover. Because if it is something that you do not know well but the label looks like what you are used to buying, you cannot tell that it is falsified by just looking at it.”* **Participant 02, Female only Farmers FGD 04, Namayumba Town Council**

There was a common perception that it was the role of a human or animal health worker to identify whether a medicine was substandard or falsified. However, this view was expressed more frequently among the farmer FGDs as many of them mentioned that they did not have the ability to know whether the medicine that was used by the veterinary doctor was substandard or not. This is because veterinary practitioners normally administered drugs directly to the animal (s) in these situations.

*“I think it is hard to know that the medicine is falsified because I am not a health worker first of all. So, when I bring a veterinary health worker to treat my animals, he/ she is the one to decide for me. I do not have the authority to convince him/ her that this medicine you have given to my animals is falsified when he/ she has explained to me that the medicine is appropriate. So, I cannot know whether the medicine is genuine or not.”* **Participant 07, Male only Farmers FGD 07, Namayumba Town Council**

### Drivers of substandard and falsified medicines

FGD participants mentioned several drivers of SFMs that were either customer or supplier driven. Customer drivers included: inadequate knowledge of SFMs, lack of finances, and lack of access to licensed drug shops/ pharmacies. Supplier drivers included limited regulation and enforcement, and the business orientation of drug shops/ pharmacies.

### Customer drivers

#### Inadequate knowledge of SFMs.

Key informants stated that most people in the community had little or no knowledge about the quality of medicines and how to make an assessment to determine if they were genuine or not. Additionally, they did not make any attempt to ascertain the integrity of a drug when making a purchase. Indeed, some community participants mentioned that they had never been educated on how to identify SFMs. Therefore, whatever medicine they were given at the point of purchase (drug shop/ pharmacy) was believed to be genuine. Other participants stated that many community members were also illiterate and therefore had to trust in the health worker providing them the medicine, as they assumed that this practitioner knew what they were doing.

*“First of all, people do not even know the type of medicine being sold to them. They are illiterate, they cannot read. They cannot even read what is on the packaging. They just come asking, I want such a drug that does this and that. When you give them the medicine, they are confident in that animal health worker who has given them the medicine. They therefore will not mind whether or not the medicine is substandard or not.”* **Key Informant 06, Veterinary personnel, Drug Shop, Kakiri Subcounty**

#### Lack of finances.

Participants emphasised that the purchasing of SFMs, for use in both humans and animals, was strongly driven by the lack of finances. Both FGD and KII participants mentioned that the purchase of medicines depended on one’s financial capabilities. Many participants stated that SFMs were generally cheaper than genuine ones yet many people could only afford the cheapest medicines. As a result, community members, especially from low-income households, were prone to buying SFMs.

*“A health worker may tell you that the medicine that would have treated your condition is around 13 USD. But when you say that you will not manage that amount of money, then the health worker will try to adjust and get medicines that will fit within your budget or the amount of money you have. So you can be given those substandard medicines because you cannot afford the genuine ones that are required.”* **Key Informant 02, Local Leader, Masulita Town Council**

#### Lack of access to licensed drug shops/ pharmacies.

FGD participants highlighted that remote and inaccessible communities were at higher risk of accessing SFMs since they were often not in the vicinity of regulated drug shops/ pharmacies for both human and animal medicines. As such, participants reported that hawkers were more commonly utilised to access medicines in these settings. Participants further noted that it was difficult for them to access designated government facilities to acquire genuine medicines because these were located a distance away hence the need for transportation. Both the CHW and farmer FGD participants also mentioned that it was difficult for them to access their local government officers (health workers and veterinary officers) and this frustration left them with no choice but to use the nearest supplier of medicines they could find.

*“On the side of veterinary officers given to us by the government at the sub-county level where we are most likely to get original medicines, even though the officers are there at the sub county, they do not come to us in the village. Because I may not have the resources, I cannot go and buy medicine from the sub-county as it is about 7 miles from my village. I thus use the sources that are closer to us*.” **Participant 01, Male only, Mixed CHWs and Farmers, FGD 06, Namayumba Town Council**

### Supplier drivers

#### Limited law regulation and enforcement.

FGD participants blamed the Government of Uganda for having weak policy regulations to monitor the distribution of SFMs on the market. Many participants stated that they did not feel confident in regulatory bodies providing regular inspections or monitoring visits for medicines assurance in their community. According to them, this weak law enforcement was exacerbated by bribery. Indeed, participants stated that many of the drug shop owners continued selling SFMs because they were able to give money as a bribe to the few inspecting and regulatory persons to allow them to continue having such drugs within their shops. Additionally, some participants felt that the regulatory and inspecting authorities were corrupt and therefore were considered promoters of the continued use of SFMs.

*“Today, some of us who buy medicines as you have heard, we know very well the one who has right and false medicine depending on the price and labelling of such medicine. However, we don’t have the authority to go and arrest those that sell substandard and falsified medicines. And even when you send there an agency that regulates medicines, the person will be bribed and when corruption happens, that medicine will remain on the market.*” **Participant 12, Male only Mixed CHWs and Farmers FGD 06, Namayumba Town Council**

#### Business orientation of drug shops/ pharmacies.

Almost all participants in the study mentioned that most people who sold medicines for human and animal use were in the business for profit and therefore hardly paid attention to whether they sold SFMs. Several scenarios of unscrupulous happenings were described by participants, including extending or changing of expiry dates for already expired medicines; adulterations of medicines for example by adding water; and intentional sale of SFMs as genuine medicines to increase the profit margin or to recover business expenses.

*“The truth is that substandard medicine also exists in human medicine and the main cause of that is greed for money like my colleagues have said. One can have many customers with limited authentic medicine so they decide to bring falsified medicine which is cheaper for them to get more customers hence more income*.” **Participant 05, Male only CHWs FGD 08, Kakiri Town Council**

### Challenges of reporting SFMs

Various challenges to reporting SFMs were identified by the participants including: not knowing where to report suspected cases of SFMs, lack of proof of purchase of SFMs, fear of reporting, and lack of trust in government procedures.

### Not knowing where to report suspected cases of SFMs

Key informants mentioned that community members were not aware of the channels of reporting if they had seen suspected or used a potential or known SFM. This was echoed in all the FGDs, as participants stated that they did not know where to report any suspected concerns relating to SFMs.

*“On how to report, even me right now, I ask myself, how do they report? How do people who do not know how to read and write report on falsified medicine? There’s no clear reporting channel, the people are not aware, both the health workers and the consumers. By the way, they really lack awareness on reporting.”* **Key informant 11, MOH Pharmacy department**

#### Lack of evidence/ proof of purchase of SFMs.

Other participants stated that reporting of SFMs required one to have evidence of sale or use of the medicine which in most cases was not readily available or accessible to them. This was because of the way medicines were dispensed to them at the point of purchase. In many drug shops/ pharmacies, medicines were bought without a receipt which made it difficult to hold the seller accountable. Therefore, complaints on any medicine were made verbally which they noted might not be credible during legal proceedings. Furthermore, many participants mentioned circumstances when the drug shop/ pharmacy sellers denied having sold them the SFMs, as they did not have tangible proof of their purchase.

*“I suppose one might not fail to get where to report substandard and falsified medicine. But most times the pharmacists do not have any record to show that he/she sold to you that medicine. When you report, they deny saying that ‘I did not sell it to you’. All accusations will be in vain because you have nothing to show that they sold you any medicine. The result is that you give up because you have no starting point.”* **Participant 06, Female only, CHWs and Farmers, FGD 10, Namayumba Sub County**

### Fear of reporting

Some FGD participants stated that they feared reporting cases of SFMs because of the lack of confidentiality when they presented their cases to the responsible persons. They stated that reporting SFMs could at times result in threats to one’s family, livelihood, relationships with colleagues, and in worse case scenarios death. This was mainly because the officials they reported to could share their personal information with the suspected SFM sellers. As a result, they preferred to keep quiet about any suspected SFMs. The release of one’s personal details could result in estranged relationships and violence in the community among the medicine sellers and their clients.

*“I have heard it happen* [the release of a reporter’s personal details] *and then people end up losing their lives. So to keep peace, some things we only look on and keep quiet. But we have heard those announcements telling us to call the number of the district for free or you can send a message, but the challenge is the reporting can put me in trouble.”* **Participant 06, Female only, CHWs FGD 02, Kakiri Town Council**

### Lack of trust in government procedures

Regarding animal health, participants mentioned that they did not trust government procedures on abating SFMs through inspections and reporting. According to many FGD participants, reporting of SFMs would not yield the desired outcome because farmers were not willing to make losses from dying animals and the government officials would not be helpful. This perspective was similar in human health, as participants mentioned that inspecting and reporting of suspected sellers of SFMs would not yield justice and that businesses would continue as usual. Such incidences were linked to reduced trust in police and government workers, particularly those working with the MOH, MAAIF, the National Drug Authority (NDA), and the National Medical Stores (NMS).

*“When someone is found red handed for example while smuggling substandard medicine, we would think that such a person should get a grave punishment to scare him from doing that again. But most times what discourages us is that when we report those people to those organisations we expect to help us, they do not punish them. You might report them to the police and even get the courage to personally take them to police. The police will show you that he/she has been detained and will be charged, but before reaching your home, they* [earlier detained person] *may reach their home before you and even sometimes show off their victory.”* **Participant 02, Male only CHWs FGD 08, Kakiri Town Council**

## Discussion

This study assessed perceptions and practices on SFMs for use in humans and animals and the potential impacts of this from a One-Health perspective in Wakiso district, Uganda. The availability of SFMs in human and animal communities is evident and the acceptance of this should not be taken lightly due to the ramifications for human, animal and environmental health [2,8,40,56]. Despite limited formal education or public health awareness campaigns, participants were able to recognise and discuss the existence of SFMs in their communities and the compounding factors of this health threat. Defining SFMs in relation to identifying their effects and appearance in both human and animal health was well described by the study participants. Studies in Sudan, Nigeria, Ethiopia, Tanzania, Lebanon and various continents have also shown that the public identifies SFMs through their side effects, appearance and packaging [49,52,59–64].

Despite being aware that SFMs could lack a quality mark, seal, batch number, label, packaging, or have a broken seal or a difference in the spelling of words on the packaging/ colour/ texture from the original, the FGD participants unanimously agreed that actual identification of SFMs was difficult without using them, as described elsewhere [52,53]. Difficulties in the identification of SFMs have also been raised in the recent WHO global report [30]. Furthermore, having knowledge about how to identify SFMs did not necessarily guarantee that community members would actually check their medicines before use, as found elsewhere [63,65]. In Nigeria, failure to verify medicines was attributed to time constraints [63]. However, in a study carried out in Tanzania, participants with knowledge of the health effects of falsified drugs better identified genuine and falsified drugs than their counterparts [64]. Our finding could be explained by the fact that participants stated that they had not been educated or trained on how to identify SFMs nor did they have the knowledge about the potential consequences on their health. Health campaigns to increase knowledge on identification of SFMs through investment in regular awareness programmes should ensure that the messages are understandable for all community members [66]. This can be achieved through using lay language without technical terminologies for the public to easily understand [67]. Use of visuals such as pictures during public health campaigns among communities with high illiteracy rates, as well as the provision of information on SFMs in local languages should also be explored.

In our study, it was reported that neither community members nor health workers knew where to report suspected SFMs. Similar findings have been found among the public [49] and health workers [68]. Enhancement of knowledge on the identification and reporting of SFMs is therefore needed, not only for the general public, but also health workers [67]. Such campaigns in Uganda should take a multisectoral approach involving various stakeholders including MOH, MAAIF, NDA, National Medical Stores (NMS), and the Pharmaceutical Society of Uganda (PSU). It is important to highlight that consumer attempts to directly report SFMs to manufacturers or question medical staff about SFMs at the point of purchase have been refuted as they are likely not to yield positive results [69], possibly due to power dynamics.

Our study showed a strong reliance on observation of the effects of SFMs after their use in humans and animals to distinguish them from genuine ones. However, this is a high-risk practice as harm caused by toxic ingredients cannot always be mitigated and may result in morbidity or death [49,70]. Whilst participants expressed safety concerns related to ineffective treatment and toxicities from SFMs, it should be noted that the public may continue to use these medicines if they fully or partially alleviate their symptoms due to their availability and affordability [52,71]. Our study participants believed it was the role of human and animal health workers to identify SFMs and stop the public from using them. In many peri-urban and rural communities, as shown by the study findings, access to medicines is usually through health and veterinary professionals, especially among farmers for their animals. Accessing medicines through trusted professionals was seen as a way to avoid SFMs, with participants placing confidence in the relationships they had cultivated with these providers, as reported in earlier studies [53,65,72,73]. However, sometimes these professionals may distribute SFMs intentionally (due to a desire to make profits/ supplement income) or unknowingly (due to a lack of knowledge on identification and reporting of SFMs) [17,30,52,53,74–76]. Additionally, a study conducted in Qatar revealed that there were few differences in levels of knowledge of SFMs between health workers and community members which is concerning [77]. Education of the public on SFMs should adopt a One Health perspective, addressing their impact across human and animal health systems, and supported by policies that encourage cross-sector collaboration. This is particularly important to reduce the risks of emergence of AMR as consumption of inadequate quantities of antimicrobials can increase the evolution of resistant micro-organisms which can be spread through the food-chain and environment. Such education should emphasize the need for animal and human medicines to be bought from their respective registered pharmacies, clinics and drug outlets.

Drivers of SFMs established in our study were divided into customer and supplier-led. Customer drivers included lack of knowledge, lack of finances, and inaccessibility of licensed drug shops/ pharmacies, which are interlinked. Recent multi-country studies in Ghana, Nigeria, Sierra Leone, and Uganda highlighted similar drivers of use of SFMs [44,66]. Our study participants believed that cheaper medicine which was thought to be substandard or falsified was more likely to reach them since the majority were residing in remote and inaccessible communities as described elsewhere [5]. Additionally, participants mentioned that their limited finances exposed them to buying cheap medicines for both humans and animals. According to the participants, their low purchasing power was linked to the likelihood of buying SFMs in the community. Similar perceptions of the cost of medicines having an impact on efficacy have been expressed in Ghana [53], Nigeria [78], Benin [79], Lebanon [65], Sudan [80] and globally [81,82]. Less privileged groups such as persons living in rural areas are likely to be disproportionally exposed to SFMs due to unaffordability of genuine medicines as shown in earlier studies [7,44,49,65] including Uganda where 97.9% of the deaths attributable to poor-quality antimalarials were among rural populations who also paid 10.7 times as much annually in out-of-pocket expenses compared with urban populations [41]. The same rural populations are more likely to be less educated, hence having limited knowledge of SFMs [44,83]. Earlier studies have raised concerns that SFMs disproportionately affect vulnerable communities, noting that their circulation is largely driven by the inaccessibility and unavailability of quality essential medicines, which in turn creates demand for cheaper alternatives exploited by SFM criminal supplier entities [18,46,71,83]. Additionally, the fear of agricultural modernisation may also expose rural veterinary communities to SFMs [84]. Such disadvantaged populations need to be prioritised while designing and implementing interventions to reduce access to and use of SFMs among the population. More importantly, the Ugandan government needs to increase household income through economic development, which would address the affordability constraints that drive poor families towards informal medicine markets. Additionally, a mechanism to subsidise the price of quality medicines could be developed so that they are more affordable to poor communities. Deploying mobile human and animal healthcare access points in remote areas would also take quality medicines closer to the population.

Our study identified poor regulation and enforcement as a supplier-led driver of SFMs which was closely linked with fear of reporting suspected cases and lack of trust in government systems. In many African countries, policy, regulation and implementation plans with respect to SFMs in human and animal health are inadequate [18,48,75,85–87]. Poor law enforcement has previously been seen to lead to corruption resulting in mistrust in government authorities and health workers [52,88]. These findings are consistent with a World Bank report that linked poor regulation and surveillance of SFMs to bribery and corruption [40]. As an example, cases of SFMs were found to have disappeared before courts of law in Uganda [40]. Studies in Ethiopia, Nigeria and Lebanon have also mentioned corruption within government and regulatory bodies to manipulate sales of SFMs for their private gain [52,89,90]. In our study, participants mentioned several incidences in which regulatory authorities were bribed by those selling SFMs to continue their businesses. Such bribery has also been described to happen among district drug inspectors in Uganda [91]. This occurrence of SFMs distributors and other personnel evading regulatory procedures has been observed in Lebanon as well [89].

Business orientation of medicine markets with the desire to make high profits has been documented in drug shops and pharmacies in Uganda [92–94]. Acts of tampering with expiry dates and alternations in medicine quantities and compositions in Uganda have also been reported for both human and animal health [39,95]. The lack of transparency and professional misconduct among medicines suppliers and government regulatory bodies regarding SFMs may be a major driver of mistrust in communities for both human and animal health [73,75,89]. While there is progress on dismantling the supply chain of SFMs through policing bodies as AFRIPOL-Interpol [96], Ugandan, government regulatory bodies, particularly the NDA, need to be more intentional in apprehending SFMs and their sellers [34]. Although pertinent recommendations for enforcing regulatory standards for quality in the production and supply of pharmaceutical products have been made [46], it is also important to note that without adequate political will and leadership, the regulation of SFMs will derail [97]. Additionally, more strict regulations and punishment for individuals or companies involved in the making, selling and distribution of SFMs is needed. For example, substantive fines for manufacturers, suspension or loss of licenses for pharmacies, clinics and drug shops, and imprisonment for fake veterinary/ human health care providers could be enforced.

Not knowing where to report suspected cases of SFMs was a recurring finding in our study as also found in Lebanon and Tanzania where the official reporting channels were unknown [89,98]. The NDA website stipulates that the public is expected to remain vigilant and report any suspected substandard products including medicines by email (druginfo@nda.or.ug) phone or text on 0800101999 (toll-free line) and +256417788100 or WhatsApp (+256740002070). Having no evidence to prove the purchase of SFMs emerged as a concern regarding reporting of SFMs. However, NDA does not require proof of purchase for one to report suspected SFMs as the post marketing surveillance team only requires specifics about the product and where it was bought, then samples are picked for quality control tests. Many participants also expressed concerns about the aftermath of exposure of their personal information upon reporting suspected SFM sellers which could lead to threats to their lives, family, friends and livelihood. This should not be the case as Uganda’s Whistleblowers Protection Act 2010 forbids the disclosure of the whistle-blowers identifying information [99]. Additionally, suspicion of SFMs can be reported anonymously to NDA using the various available channels including telephone and standard forms. To encourage reporting of suspected SFMs without fear of the consequences among the public including CHWs and farmers, increasing awareness on the anonymous channels such as phone hotlines and websites that can be used is needed. Establishment of a drug tracking code system based on barcode or two-dimensional code so that each drug could be traced throughout its supply chain to its end user may also be explored. Mandatory transaction receipts for pharmaceutical products during pharmacy and drug shop sales would provide a good starting point as proof for investigating SFM cases.

The study was undertaken in Wakiso district which might limit the generalizability of findings to other regions of Uganda with different demographics, healthcare access, or agricultural practices. Since self-reported data was collected, some participants especially human and animal health practitioners could have provided some information with a bias to appear correct since it is assumed that health professionals exhibit appropriate practices regarding SFMs. A strength of this study is that it was conducted among various stakeholders beyond health practitioners including policy makers, CHWs, farmers, and local leaders through both FGDs and KIIs. To gather diverse perspectives on SFMs, we carefully selected study participants including the separation of FGDs by gender, age and role, thereby incorporating gender, cultural and social considerations. While consistent with previous suggestions [8,29,30,46,78,83,86], our recommendations extend these by emphasising community-level and One health perspectives.

## Conclusions

Several drivers of SFMs in the community among both humans and animals were established related to individuals such as a lack of knowledge, sources of medicines particularly drug shops and pharmacies, and weak regulatory frameworks including limited enforcement by the authorities. The use of the One Health approach showed the need of a unified approach to address SFMs as the experiences and challenges in human and animal health were similar. Increased awareness and better enforcement of regulations are needed to reduce the use and risks of SFMs in the community. Campaigns on visual identification and clear reporting channels for SFMs should be strengthened to protect public health and improve patient outcomes.

## Supporting information

S1 Dataset This is the study dataset. (DOCX)

S1 File Substandard and Falsified Medicines in humans and animals in Wakiso district, Uganda: a qualitative study. (DOCX)
