# Supplementary material for: Perceptions and practices on substandard and falsified medicines in humans and animals in Wakiso district, Uganda: A qualitative study
Source: PLoS One. 2025 Dec 26;20(12):e0339569. doi: 10.1371/journal.pone.0339569 (PMC12742756; doi:10.1371/journal.pone.0339569)
Supplement: S1 File — (DOCX) [file pone.0339569.s002.docx]

**Substandard and Falsified Medicines in humans and animals in Wakiso district, Uganda: a qualitative study**

| Name | Description | Files | References |
| --- | --- | --- | --- |
| **Consequences of SFMs** |  | 0 | 0 |
| AMR |  | 3 | 6 |
| Death |  | 14 | 38 |
| Disability |  | 6 | 14 |
| Get other diseases |  | 16 | 38 |
| Increased expenditure-poverty |  | 8 | 20 |
| Loss of trust in the system |  | 8 | 9 |
| Loss of yields and poor quality |  | 7 | 12 |
| Prolonged illness |  | 6 | 10 |
| Side effects poisoning |  | 8 | 11 |
| **Differentiating / identification of SFMs** |  | 0 | 0 |
| Ask professional person |  | 6 | 7 |
| Colour |  | 8 | 12 |
| Expiry date |  | 10 | 13 |
| Label and batch number |  | 10 | 19 |
| Read manual |  | 1 | 1 |
| Seal and package |  | 11 | 27 |
| Stamp |  | 5 | 11 |
| Texture of medicine powder | Mainly powder, metallic | 8 | 10 |
| **Trust & belief health workers should identify SFMS** |  | 15 | 37 |
| **Drivers of SFMS** |  | 0 | 0 |
| Accessibility- being far from services |  | 10 | 18 |
| Advertisement |  | 7 | 7 |
| Buyers’ poverty & low cost of SFMS (D) |  | 21 | 55 |
| Can’t tell or limited awareness on SFMs |  | 0 | 0 |
| Limited awareness (D) |  | 19 | 52 |
| Not easy to identify (D) |  | 15 | 78 |
| Demand |  | 4 | 10 |
| Inadequate knowledge on the implications & consequences of SFMS (D) |  | 11 | 16 |
| Malicious, corruption and nepotism (S) |  | 0 | 0 |
| Bribe & corruption(S) |  | 9 | 27 |
| Government negligence poor surveillance (S) |  | 7 | 13 |
| Self-medication (D) |  | 5 | 9 |
| Sellers' poverty vs profit (S) | Business orientation | 12 | 29 |
|  |  |  |  |
| Unqualified personnel (S) |  | 11 | 24 |
| **More SFMS medicines are more in** |  | 0 | 0 |
| Equal |  | 10 | 13 |
| More animals |  | 12 | 21 |
| More crops |  | 6 | 11 |
| More humans |  | 6 | 7 |
| No use of SFM in both humans and animals |  | 12 | 61 |
| **Challenges of reporting on SFMS** |  | 0 | 0 |
| Bribes |  | 12 | 34 |
| Don't know where to report | Unawareness of reporting channels | 19 | 51 |
| Fear to report | Due life threats, no confidentiality, worry about safety | 16 | 38 |
| No proof / evidence |  | 7 | 20 |
| No trust - Cheaters, lies and negligence |  | 16 | 33 |
| **Sources of SFMS** |  | 17 | 40 |
| Examples of SFMS |  | 6 | 9 |
| **Understanding of SFMS/ Definition of SFMs** | Definition of SFMS | 0 | 0 |
| Alternations at the point of care |  | 7 | 13 |
| Cheaper than others |  | 6 | 10 |
| Colour |  | 1 | 1 |
| Disability and death |  | 6 | 9 |
| Expired medicines |  | 14 | 34 |
| Hawks |  | 4 | 7 |
| Side effects and bad reactions to the body |  | 11 | 29 |
| Storage of medicine |  | 8 | 12 |
| Traditional medicine |  | 2 | 2 |
| True definition |  | 7 | 12 |
| Using different mixing amounts |  | 6 | 10 |
| Worse or no change in patient |  | 20 | 76 |
| Wrong diagnosis versus medicine given |  | 7 | 9 |
